# Supplementary material for: Hydrodynamics of a twisting slender swimmer
Source: R Soc Open Sci. 2020 Aug 5;7(8):200754. doi: 10.1098/rsos.200754 (PMC7481709; doi:10.1098/rsos.200754)
Supplement: An extension [file rsos200754supp1.pdf]

Supplementary material to

# Hydrodynamics of a twisting slender swimmer

## The case of three propulsion waves

Gil Iosilevskii and Alexander Rashkovsky

Consider the case where three propulsive harmonic waves

$$y_0(t,x)=\hat{y}_0(x)+\hat{v}_0(x)\cos\left(\omega t-\kappa x+\phi_y\right), \tag{S1}$$

$$z_0(t,x)=\hat{z}_0(x)\cos(\omega t-\kappa x+\phi_z), \tag{S2}$$

$$\theta(t,x)=\hat{\theta}_0(x)\cos(\omega t-\kappa x+\phi_\theta), \tag{S3}$$

having the same wave number $\kappa$ and the same frequency $\omega$, simultaneously propagate along the body of the swimmer, each with its respective phase angle $\phi_y$, $\phi_z$ and $\phi_\theta$. Here, $\hat{v}_0$, $\hat{z}_0$ and $\hat{\theta}_0$ are the modulating amplitudes, and $\hat{y}_0$ is the time averaged bend of the swimmer's body in the vertical plane. With these, the relevant expressions for the time averaged forces follow from equations (3.26)-(3.34) in the companion paper:

$$\begin{aligned}\frac{T}{\pi s_t^2}=&\frac{1}{8}\Big(1+J_0\big(2\hat{\theta}_t\big)+J_2\big(2\hat{\theta}_t\big)\cos 2\big(\phi_z-\phi_\theta\big)\Big)\Big(\big(\omega^2-\kappa^2\big)\hat{z}_t^2-\dot{\hat{z}}_t^2\Big)+\frac{s_t^2}{32}\Big(\big(\omega^2-\kappa^2\big)\hat{\theta}_t^2-\dot{\hat{\theta}}_t^2\Big)\\&+\frac{1}{8}\Big(1-J_0\big(2\hat{\theta}_t\big)-J_2\big(2\hat{\theta}_t\big)\cos 2\big(\phi_y-\phi_\theta\big)\Big)\Big(\big(\omega^2-\kappa^2\big)\hat{v}_t^2-\dot{\hat{v}}_t^2\Big)-\frac{1}{4}\Big(1-J_0\big(2\hat{\theta}_t\big)\Big)\dot{\hat{y}}_t^2\\&+\frac{1}{4}J_2\big(2\hat{\theta}_t\big)\Big(\dot{\hat{z}}_t^2\cos 2\big(\phi_z-\phi_\theta\big)+\kappa\dot{\hat{z}}_t\hat{z}_t\sin 2\big(\phi_z-\phi_\theta\big)\Big)\\&-\frac{1}{4}J_2\big(2\hat{\theta}_t\big)\Big(\dot{\hat{v}}_t^2\cos 2\big(\phi_y-\phi_\theta\big)+\kappa\dot{\hat{v}}_t\hat{v}_t\sin 2\big(\phi_y-\phi_\theta\big)\Big)\\&+\frac{1}{2}J_1\big(2\hat{\theta}_t\big)\dot{\hat{y}}_t\Big(\hat{z}_t\kappa\sin\big(\phi_z-\phi_\theta\big)+\dot{\hat{z}}_t\cos\big(\phi_z-\phi_\theta\big)\Big),\end{aligned} \tag{S4}$$

$$\frac{L}{\pi s_t^2}=\frac{1}{2}J_1\big(2\hat{\theta}_t\big)\Big(-\hat{z}_t(\omega-\kappa)\sin\big(\phi_z-\phi_\theta\big)+\dot{\hat{z}}_t\cos\big(\phi_z-\phi_\theta\big)\Big)-\frac{1}{2}\Big(1-J_0\big(2\hat{\theta}_t\big)\Big)\dot{\hat{y}}_t, \tag{S5}$$

$$\frac{Z}{\pi s_t^2} = \frac{1}{2} J_1\left(2\hat{\theta}_t\right)\left(-\hat{v}_t\,(\omega-\kappa)\sin\left(\phi_y-\phi_\theta\right)+\dot{\hat{v}}_t\cos\left(\phi_y-\phi_\theta\right)\right), \tag{S6}$$

$$\frac{P}{\pi s_t^2} = \frac{1}{4}\omega(\omega-\kappa)\left(\hat{z}_t^2\left(1+J_0\left(2\hat{\theta}_t\right)+J_2\left(2\hat{\theta}_t\right)\cos 2\left(\phi_z-\phi_\theta\right)\right)+\frac{s_t^2\hat{\theta}_t^2}{4}\right)$$

$$+\frac{1}{4}\omega(\omega-\kappa)\hat{v}_t^2\left(1-J_0\left(2\hat{\theta}_t\right)-J_2\left(2\hat{\theta}_t\right)\cos 2\left(\phi_y-\phi_\theta\right)\right)$$

$$+\frac{1}{4}\omega\left(\hat{z}_0\dot{\hat{z}}_0 J_2\left(2\hat{\theta}_t\right)\sin 2\left(\phi_z-\phi_\theta\right)-\hat{v}_t\dot{\hat{v}}_t J_2\left(2\hat{\theta}_t\right)\sin 2\left(\phi_y-\phi_\theta\right)\right)$$

$$+\frac{1}{2}\omega J_1\left(2\hat{\theta}_t\right)\hat{z}_t\dot{\hat{y}}_t\sin\left(\phi_z-\phi_\theta\right), \tag{S7}$$

$$\frac{M_{x'}}{\pi s_t^2} = -\frac{1}{2}(\omega-\kappa)\,\hat{y}_t\hat{v}_t\sin\left(\phi_y-\phi_\theta\right)J_1\left(2\hat{\theta}_t\right)+\frac{1}{2}\left(\dot{\hat{y}}_t\hat{v}_t+\hat{y}_t\dot{\hat{v}}_t\right)J_1\left(2\hat{\theta}_t\right)\cos\left(\phi_y-\phi_\theta\right)$$

$$-\frac{1}{2}(\omega-\kappa)\,\hat{v}_t\hat{z}_t\left(\sin\left(\phi_y-\phi_z\right)+J_2\left(2\hat{\theta}_t\right)\sin\left(\phi_y+\phi_z-2\phi_\theta\right)\right)$$

$$-\frac{1}{4}\left(\dot{\hat{z}}_t\hat{v}_t+\dot{\hat{v}}_t\hat{z}_t\right)\left(J_0\left(2\hat{\theta}_t\right)\cos\left(\phi_y-\phi_z\right)-\cos\left(\phi_y+\phi_z-2\phi_\theta\right)J_2\left(2\hat{\theta}_t\right)\right)$$

$$-\frac{1}{4}\left(\dot{\hat{z}}_t\hat{v}_t-\dot{\hat{v}}_t\hat{z}_t\right)\cos\left(\phi_y-\phi_z\right), \tag{S8}$$

$$\frac{M_{z',t}}{\pi s_t^2} = \frac{1}{2s_t^2}\int_{x_n}^{x_t} s^2(x)\left(\hat{z}_0(x)(\omega-\kappa)\sin\left(\phi_z-\phi_\theta\right)-\dot{\hat{z}}_0(x)\cos\left(\phi_z-\phi_\theta\right)\right)J_1\left(2\hat{\theta}_0(x)\right)dx$$

$$+\frac{1}{2s_t^2}\int_{x_n}^{x_t} s^2(x)\,\dot{\hat{y}}_0(x)\left(1-J_0\left(2\hat{\theta}_0(x)\right)\right)dx\,. \tag{S9}$$

Subscript ‘$t$’ marks the value of the respective function at the caudal end. There is an obvious similarity between the effects of in-plane (associated with $\hat{v}_0$) and out-of-plane (associated with $\hat{z}_0$) lateral waves, but there is no identity. Suffice it to say that the effect of the in-plane oscillations vanishes with $\hat{\theta}_t = \hat{\theta}_0(x_t)$, whereas the effect of the out-of-plane oscillations clearly remains. Except for $M_{x'}$, there is no interaction between the waves.
