# Supplementary material for: Hydrodynamics of a twisting slender swimmer
Source: R Soc Open Sci. 2020 Aug 5;7(8):200754. doi: 10.1098/rsos.200754 (PMC7481709; doi:10.1098/rsos.200754)
Supplement: A verification [file rsos200754supp2.pdf]

Supplementary material to

Hydrodynamics of a twisting slender swimmer

A verification

Gil Iosilevskii and Alexander Rashkovsky

The quantities shown on the following figures are the integrals

$$T(t) = -\int_{x_n}^{x_t} f_{x'}(t,x)\,dx, \tag{S1}$$

$$L(t) = \int_{x_n}^{x_t} f_{y'}(t,x)\,dx, \tag{S2}$$

$$Z(t) = \int_{x_n}^{x_t} f_{z'}(t,x)\,dx, \tag{S3}$$

$$P(t) = \int_{x_n}^{x_t} \iota(t,x)\,dx, \tag{S4}$$

$$M_{x'}(t) = \int_{x_n}^{x_t} m_{x'}(t,x)\,dx, \tag{S5}$$

$$M_{y',t}(t) = -\int_{x_n}^{x_t} f_{z'}(t,x)(x - x_t)\,dx, \tag{S6}$$

$$M_{z',t}(t) = \int_{x_n}^{x_t} f_{y'}(t,x)(x - x_t)\,dx \tag{S7}$$

representing instantaneous thrust, lift, side force, power, and three components of the hydrodynamic moment referred to the caudal end. The integrands in (S1)-(S7) can be found in equations (3.10), (3.7), (3.8), (3.23), (3.18), (3.8) and (3.7) of the companion paper. Exploiting equations (2.17) and (2.18) ibid., as well as the assumption that $s(x_n) = s_n = 0$, equations (S1) -(S5) can be recast as

$$T=\frac{\pi}{2}\left(s^2\left(\left(\cos\theta\frac{\partial z_0}{\partial t}-\sin\theta\frac{\partial y_0}{\partial t}\right)^2-\left(\cos\theta\frac{\partial z_0}{\partial x}-\sin\theta\frac{\partial y_0}{\partial x}\right)^2\right)+\frac{s^4}{8}\left(\left(\frac{\partial\theta}{\partial t}\right)^2-\left(\frac{\partial\theta}{\partial x}\right)^2\right)\right)_{x=x_t}$$

$$-\pi\frac{\partial}{\partial t}\int_{x_n}^{x_t}\left(s^2\left(\cos\theta\frac{Dz_0}{Dt}-\sin\theta\frac{Dy_0}{Dt}\right)\left(\cos\theta\frac{\partial z_0}{\partial x}-\sin\theta\frac{\partial y_0}{\partial x}\right)+\frac{s^4}{8}\frac{D\theta}{Dt}\frac{\partial\theta}{\partial x}\right)dx\,, \tag{S8}$$

$$L=\frac{\pi}{2}\left(s^2\left(\sin 2\theta\frac{Dz_0}{Dt}-2\sin^2\theta\frac{Dy_0}{Dt}\right)\right)_{x=x_t}+\frac{\pi}{2}\frac{\partial}{\partial t}\int_{x_n}^{x_t}s^2\left(\sin 2\theta\frac{Dz_0}{Dt}-2\sin^2\theta\frac{Dy_0}{Dt}\right)dx\,, \tag{S9}$$

$$Z=-\frac{\pi}{2}\left(s^2\left(2\cos^2\theta\frac{Dz_0}{Dt}-\sin 2\theta\frac{Dy_0}{Dt}\right)\right)_{x=x_t}-\frac{\pi}{2}\frac{\partial}{\partial t}\int_{x_n}^{x_t}s^2\left(2\cos^2\theta\frac{Dz_0}{Dt}-\sin 2\theta\frac{Dy_0}{Dt}\right)dx\,, \tag{S10}$$

$$P=\pi\left(s^2\left(-\cos\theta\frac{Dz_0}{Dt}+\sin\theta\frac{Dy_0}{Dt}\right)\left(-\cos\theta\frac{\partial z_0}{\partial t}+\sin\theta\frac{\partial y_0}{\partial t}\right)+\frac{s^4}{8}\frac{D\theta}{Dt}\frac{\partial\theta}{\partial t}\right)_{x=x_t}$$

$$+\frac{\pi}{2}\frac{\partial}{\partial t}\int_{x_n}^{x_t}s^2\left(\left(\cos\theta\frac{\partial z_0}{\partial t}-\sin\theta\frac{\partial y_0}{\partial t}\right)^2-\left(\cos\theta\frac{\partial z_0}{\partial x}-\sin\theta\frac{\partial y_0}{\partial x}\right)^2\right)dx$$

$$+\frac{\pi}{16}\frac{\partial}{\partial t}\int_{x_n}^{x_t}s^4\left(\left(\frac{\partial\theta}{\partial t}\right)^2-\left(\frac{\partial\theta}{\partial x}\right)^2\right)dx\,, \tag{S11}$$

$$M_{x'}=\pi\left(s^2\left(z_0\sin\theta+y_0\cos\theta\right)\left(-\cos\theta\frac{Dz_0}{Dt}+\sin\theta\frac{Dy_0}{Dt}\right)-\frac{s^4}{8}\frac{D\theta}{Dt}\right)_{x=x_t}$$

$$+\pi\frac{\partial}{\partial t}\int_{x_n}^{x_t}\left(s^2\left(z_0\sin\theta+y_0\cos\theta\right)\left(-\cos\theta\frac{Dz_0}{Dt}+\sin\theta\frac{Dy_0}{Dt}\right)-\frac{s^4}{8}\frac{D\theta}{Dt}\right)dx\,, \tag{S12}$$

where, hoping that no confusion could result, the arguments of all functions have been abbreviated. Averaging (S8)-(S12) with respect to time recovers explicit forms of equations (3.26), (3.27), (3.28), (3.29) and (3.30) in the companion paper:

$$\langle T\rangle=\frac{\pi}{2}\left(s^2\left\langle\left(\cos\theta\frac{\partial z_0}{\partial t}-\sin\theta\frac{\partial y_0}{\partial t}\right)^2-\left(\cos\theta\frac{\partial z_0}{\partial x}-\sin\theta\frac{\partial y_0}{\partial x}\right)^2\right\rangle+\frac{s^4}{8}\left\langle\left(\frac{\partial\theta}{\partial t}\right)^2-\left(\frac{\partial\theta}{\partial x}\right)^2\right\rangle\right)_{x=x_t}, \tag{S13}$$

$$\langle L\rangle=\frac{\pi}{2}\left(s^2\left\langle\sin 2\theta\frac{Dz_0}{Dt}-2\sin^2\theta\frac{Dy_0}{Dt}\right\rangle\right)_{x=x_t}, \tag{S14}$$

$$\langle Z\rangle=-\frac{\pi}{2}\left(s^2\left\langle 2\cos^2\theta\frac{Dz_0}{Dt}-\sin 2\theta\frac{Dy_0}{Dt}\right\rangle\right)_{x=x_t}, \tag{S15}$$

$$\langle P\rangle = \pi\left(s^2\left\langle\left(-\cos\theta\frac{Dz_0}{Dt}+\sin\theta\frac{Dy_0}{Dt}\right)\left(-\cos\theta\frac{\partial z_0}{\partial t}+\sin\theta\frac{\partial y_0}{\partial t}\right)\right\rangle+\frac{s^4}{8}\left\langle\frac{D\theta}{Dt}\frac{\partial\theta}{\partial t}\right\rangle\right)_{x=x_t}, \tag{S16}$$

$$\langle M_{x'}\rangle = \pi\left(s^2\left\langle\left(z_0\sin\theta+y_0\cos\theta\right)\left(-\cos\theta\frac{Dz_0}{Dt}+\sin\theta\frac{Dy_0}{Dt}\right)\right\rangle\right)_{x=x_t}. \tag{S17}$$

The aim of this supplementary is to establish the coherence of equations (S6)-(S12) under the assumptions of the slender body theory. We do so by comparison with numerical simulations based on the vortex lattice method [1]. The particular implementation of this method was based on vortex ring elements; it followed the paradigm described in [1], practically to a point. The code was carefully corroborated on a few previous occasions [2, 3]. The particular cases chosen for comparison were based on

$$z_0(t,x) = \hat{z}_t\bar{z}_0(x)\cos\left(\omega t-\kappa\frac{x-x_t}{x_t-x_n}\right), \tag{S18}$$

$$\theta(t,x) = \hat{\theta}_t\bar{\theta}_0(x)\cos\left(\omega t-\kappa\frac{x-x_t}{x_t-x_n}+\phi_\theta\right), \tag{S19}$$

$$y_0(t,x) = \hat{y}_t\bar{y}_0(x), \tag{S20}$$

$$s(x) = s_t\bar{s}(x), \tag{S21}$$

where

$$\bar{z}_0(x) = \bar{\theta}_0(x) = \frac{x-x_n}{x_t-x_n}-\frac{1}{2}\left(\frac{x-x_n}{x_t-x_n}\right)^2, \tag{S22}$$

$$\bar{y}_0(x) = \left(\frac{x-x_n}{x_t-x_n}\right)^2, \tag{S23}$$

$$\bar{s}(x) = \left(\frac{x-x_n}{x_t-x_n}\right)^m, \tag{S24}$$

and the constants ω, κ, $\hat{z}_t$, $\hat{\theta}_t$, ϕ_θ, $\hat{y}_t$, s_t, m, x_n and x_t are specified in Table S1 below. With $m = 1/2$, these are variants of case 1 of Table 3 in the companion paper (see Fig. S0).

Table S1: Simulation cases. The number of a case is also the number of the figure on which it is displayed.

case	$\omega/2\pi$	$\kappa/2\pi$	$\hat{z}_t$	$\hat{\theta}_t$ deg	ϕ_θ deg	$\hat{y}_t$	$1/s_t$	x_t	m
1	0.5	0.25	0.025	0	0	0	20	0	0.5
2	0.5	0.25	0.025	45	90	0	20	0	0.5
3	0.5	0.25	0.025	45	0	0	20	0	0.5
4	0.5	0.25	0.025	80	90	0	20	0	0.5
5	0.5	0.25	0.025	80	0	0	20	0	0.5
6	0.5	0.25	0.025	45	90	0.012	20	0	0.5
7	0.5	0.25	0.025	45	0	0.012	20	0	0.5
8	0.5	0.25	0.025	80	90	0.012	20	0	0.5
9	0.5	0.25	0.025	80	0	0.012	20	0	0.5
10	0.5	0.25	0.05	45	90	0	20	0	0.5
11	0.5	0.25	0.05	45	0	0	20	0	0.5
12	0.75	0.5	0.05	45	90	0	20	0	0.5
13	0.75	0.5	0.05	45	0	0	20	0	0.5
14	1	0.75	0.05	0	0	0	20	0	0.5
15	1	0.75	0.05	45	90	0	20	0	0.5
16	1	0.75	0.05	45	0	0	20	0	0.5
17	1	0.75	0.05	0	0	0	20	0	1
18	1	0.75	0.05	45	90	0	20	0	1
19	1	0.75	0.05	45	0	0	20	0	1

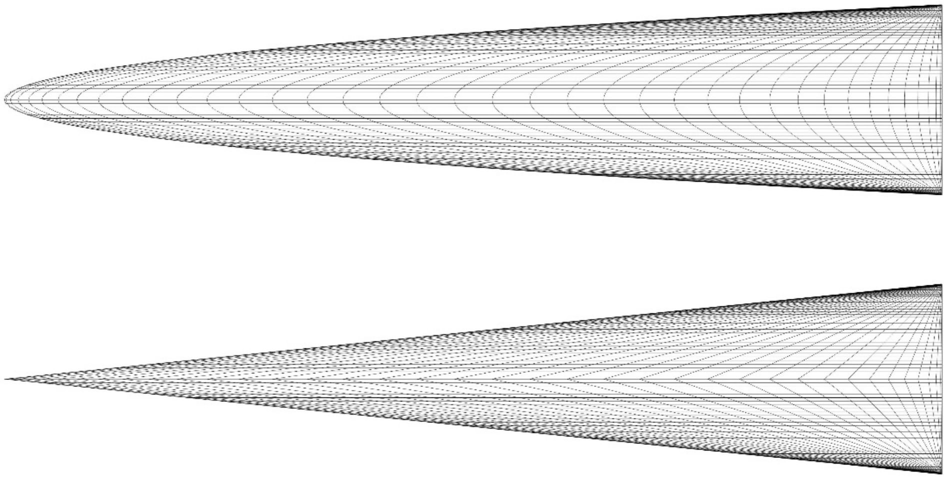

Figure S0: Cases $m = 1/2$ (top) and $m = 1$ (bottom) and their respective simulation grids. Lateral dimensions have been doubled here to make the grids visible.

The body of the swimmer was divided into 40 length-wise and 80 span-wise cells (Fig. S0), which turned out to be a good compromise between accuracy and execution time. Simulations continued for 40 steps, extending two steps more than a single period. The following figures display all simulation steps but the first and the last. Because there is practically no wake influence on a swimming slender body (as manifested in equivalence of the first and last simulation steps), the roll-up of the wake into concentrated vortices was inhibited; however, the wake did follow the trace of the trailing edge.

As long as body angles relative to the swimming direction are small, and no significant part of the upper and lower edges trails during a tail-beat, the agreement between the theory and the respective numerical simulations is excellent (cases 1-11). One must conclude that the theory is coherent under its assumptions. The difference between the theory and the simulations increases as these assumptions become increasingly abused (cases 12-19). It is the smallness of body angles relative to the swimming direction that is violated in cases 14 and 17; it is both the smallness of body angles and the windward orientation of body edges that are violated in cases 12, 13, 15, 16, 18 and 19. Whilst the smallness of body angles is an assumption underlying only the slender body theory (but not the numerical simulations), the windward orientation of body edges is an assumption underlying the slender body theory and the numerical simulations alike. Its violation renders the numerical simulations inadequate to establish the applicability limits of the slender body theory. These limits will have to be established by comparison with either carefully laid experiments or high-fidelity (e.g. RANS) numerical simulations, which are free of any of the assumptions underlying the slender body theory.

The units of forces, moments and power shown on the figures were changed from $\rho v^2 l^2$, $\rho v^2 l^3$ and $\rho v^3 l^2$ (which were used in the companion paper; $l = x_t - x_n$) to $\rho v^2 s_t^2$, $\rho v^2 s_t^3$ and $\rho v^3 s_t^2$ (where $s_t = s\left(x_t\right)$ is the semi-span at the caudal end). It is reminded that positive directions of the pitching ($M_{z'}$) and rolling ($M_{x'}$) moments are conformal with the

directions of the respective axes - they are positive when pitching down and rolling left.

References

[1] Katz J. and Plotkin A., *Low speed aerodynamics*, McGraw Hill, 1991: 479-486

[2] Iosilevskii G., “Forward flight of birds revisited. Part 1: Aerodynamics and performance,” *Royal Society Open Scienc*, 2014, **1**(2)

[3] Iosilevskii G., 2014, “Hydrodynamics of the undulatory swimming gait of batoid fishes,” *European Journal of Mechanics - B/Fluids*, 2014, **45**:12–19.

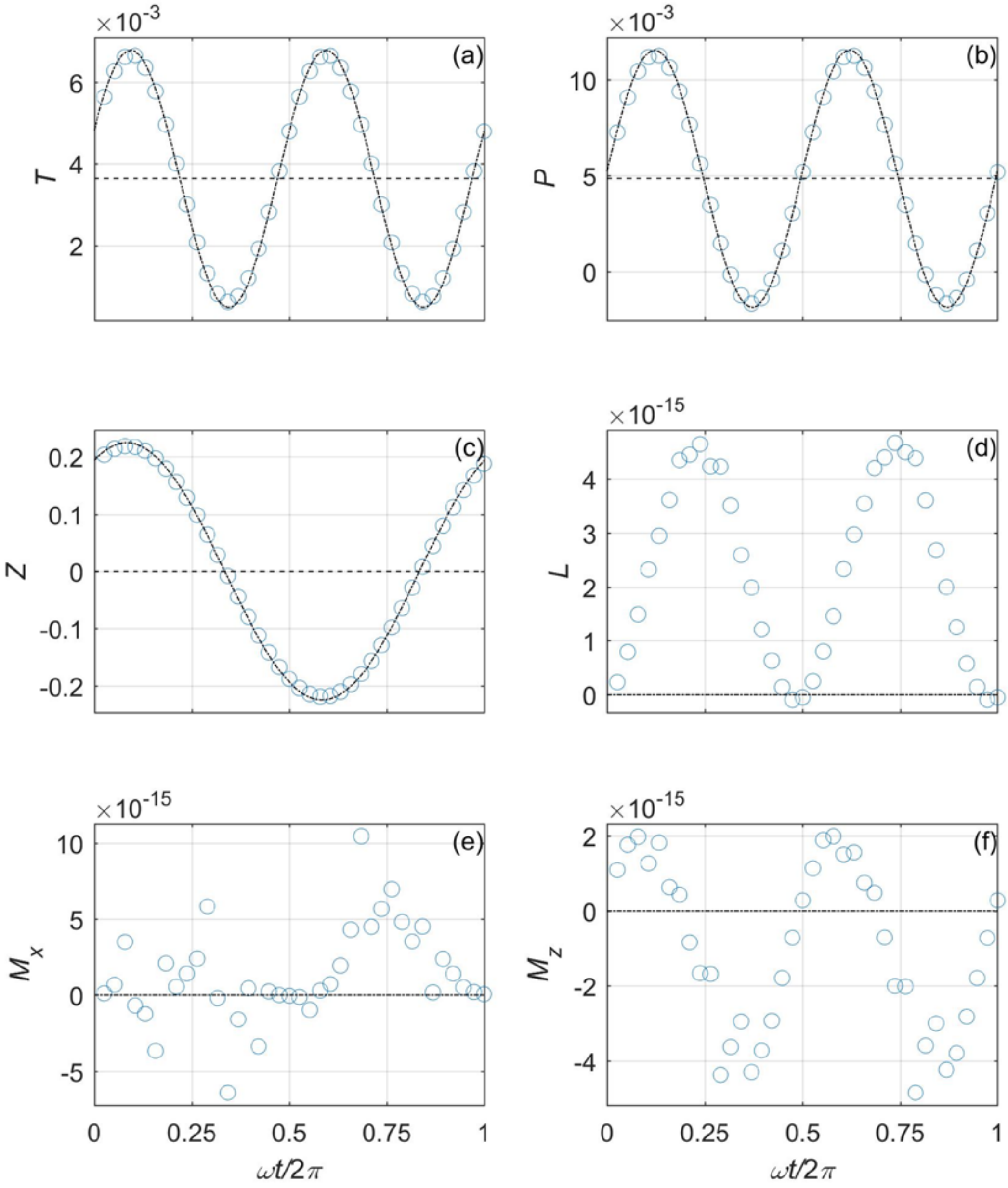

Figure S1: T, P, Z, L, $M_{x'}$ and $M_{z',t}$ over a single period. Circles mark the respective numerical simulation; dash-dot lines show equations (S8), (S11), (S10), (S9), (S12) and (S7), respectively; horizontal dashed lines mark the time-averaged values from (3.26), (3.29), (3.28), (3.27), (3.30) and (3.34) from the companion paper. Case 1 from Table S1. Note the diminutive values of L, $M_{x'}$ and $M_{z',t}$

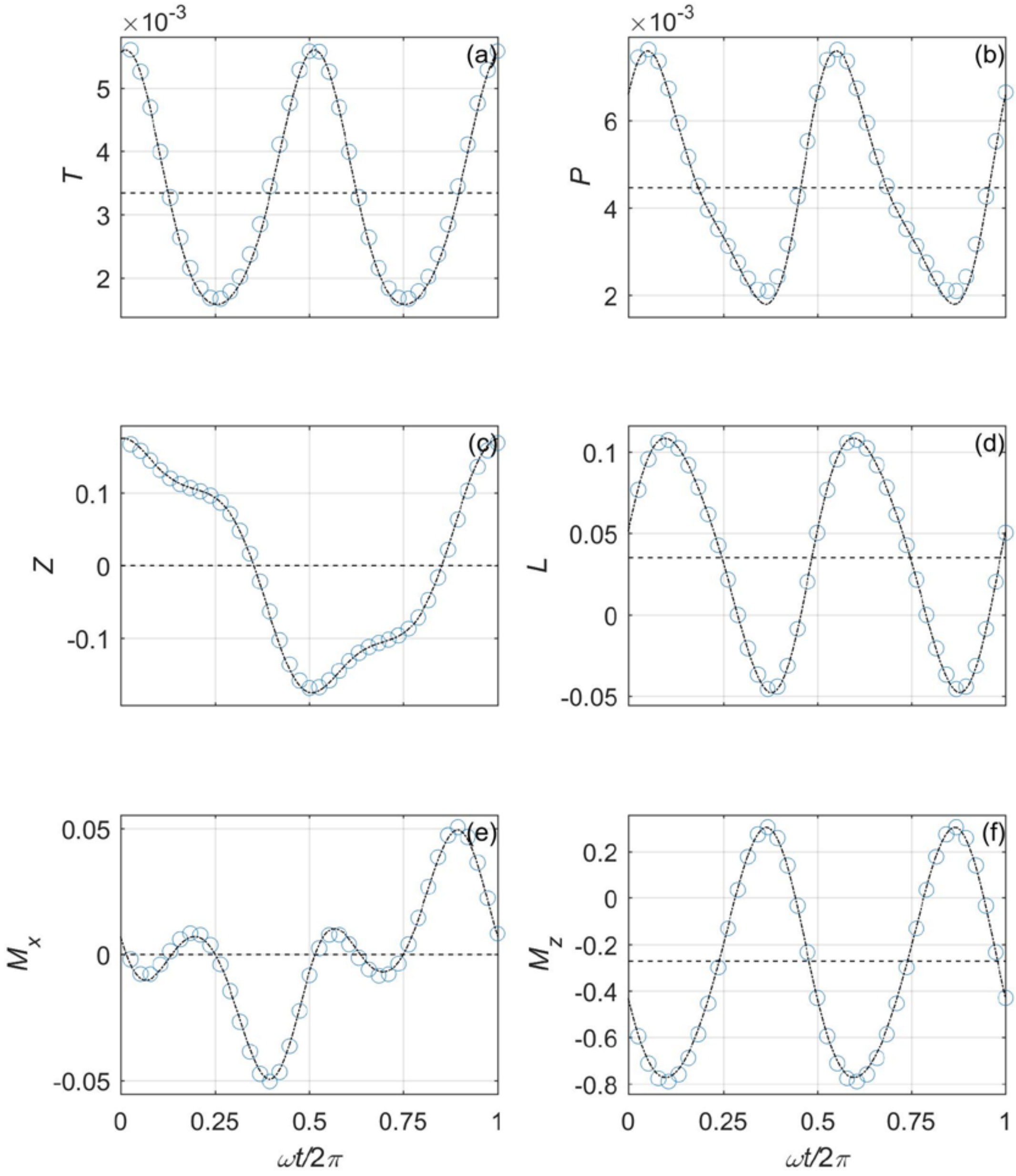

Figure S2: T, P, Z, L, $M_{x'}$ and $M_{z',t}$ over a single period. Circles mark the respective numerical simulation; dash-dot lines show equations (S8), (S11), (S10), (S9), (S12) and (S7), respectively; horizontal dashed lines mark the time-averaged values from (3.26), (3.29), (3.28), (3.27), (3.30) and (3.34) from the companion paper. Case 2 from Table S1.

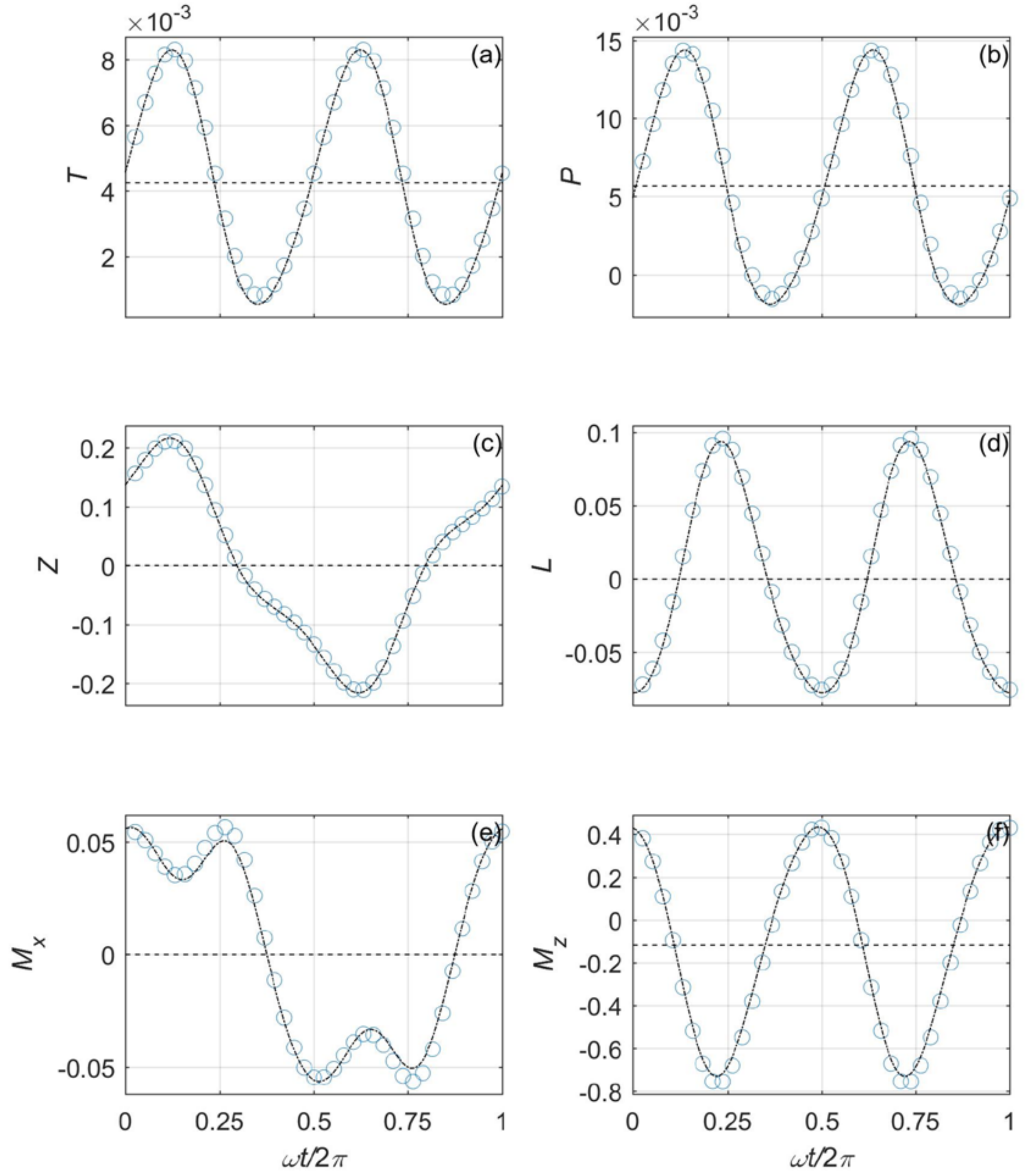

Figure S3: T, P, Z, L, $M_{x'}$ and $M_{z',t}$ over a single period. Circles mark the respective numerical simulation; dash-dot lines show equations (S8), (S11), (S10), (S9), (S12) and (S7), respectively; horizontal dashed lines mark the time-averaged values from (3.26), (3.29), (3.28), (3.27), (3.30) and (3.34) from the companion paper. Case 3 from Table S1.

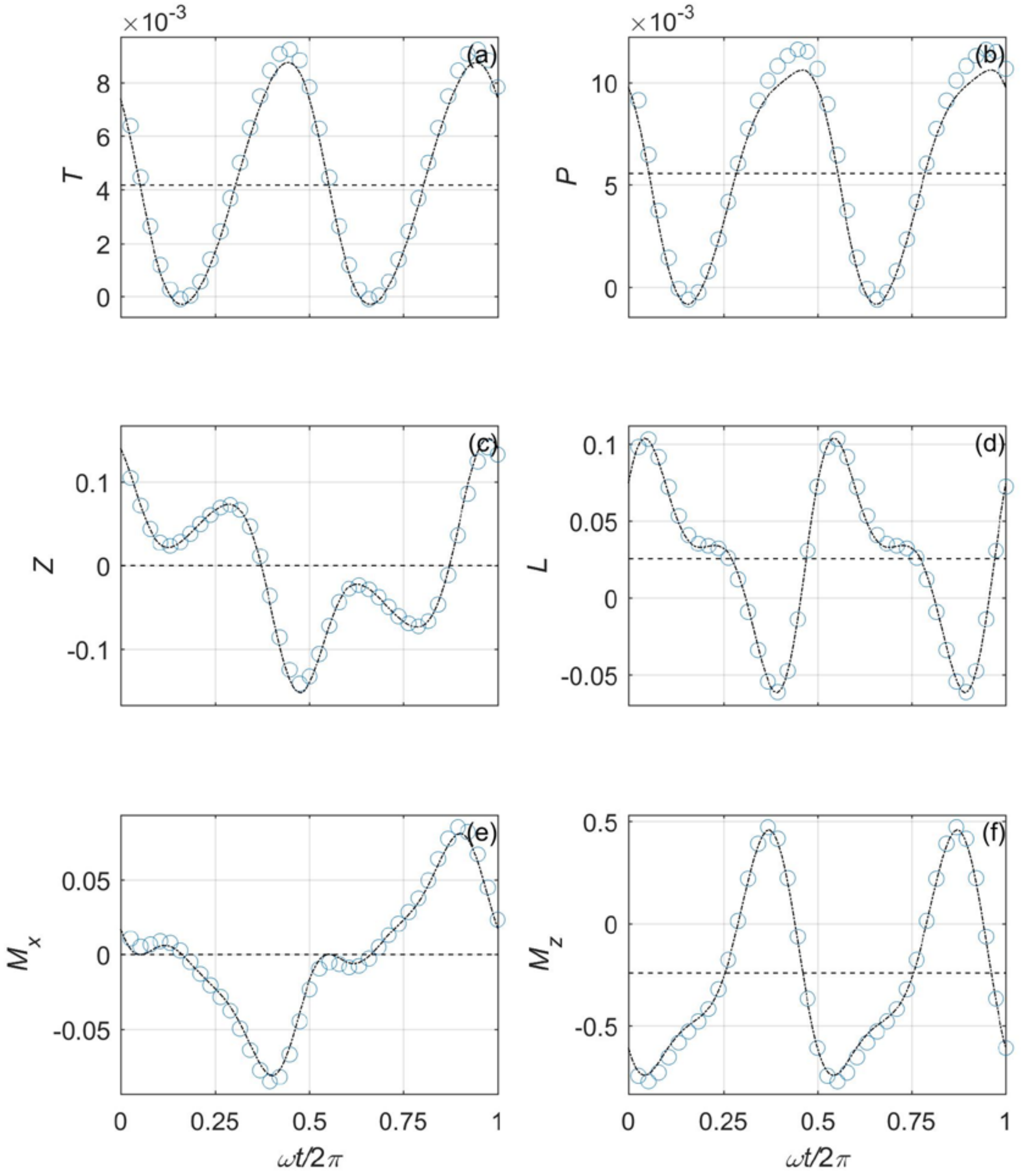

Figure S4: T, P, Z, L, $M_{x'}$ and $M_{z',t}$ over a single period. Circles mark the respective numerical simulation; dash-dot lines show equations (S8), (S11), (S10), (S9), (S12) and (S7), respectively; horizontal dashed lines mark the time-averaged values from (3.26), (3.29), (3.28), (3.27), (3.30) and (3.34) from the companion paper. Case 4 from Table S1.

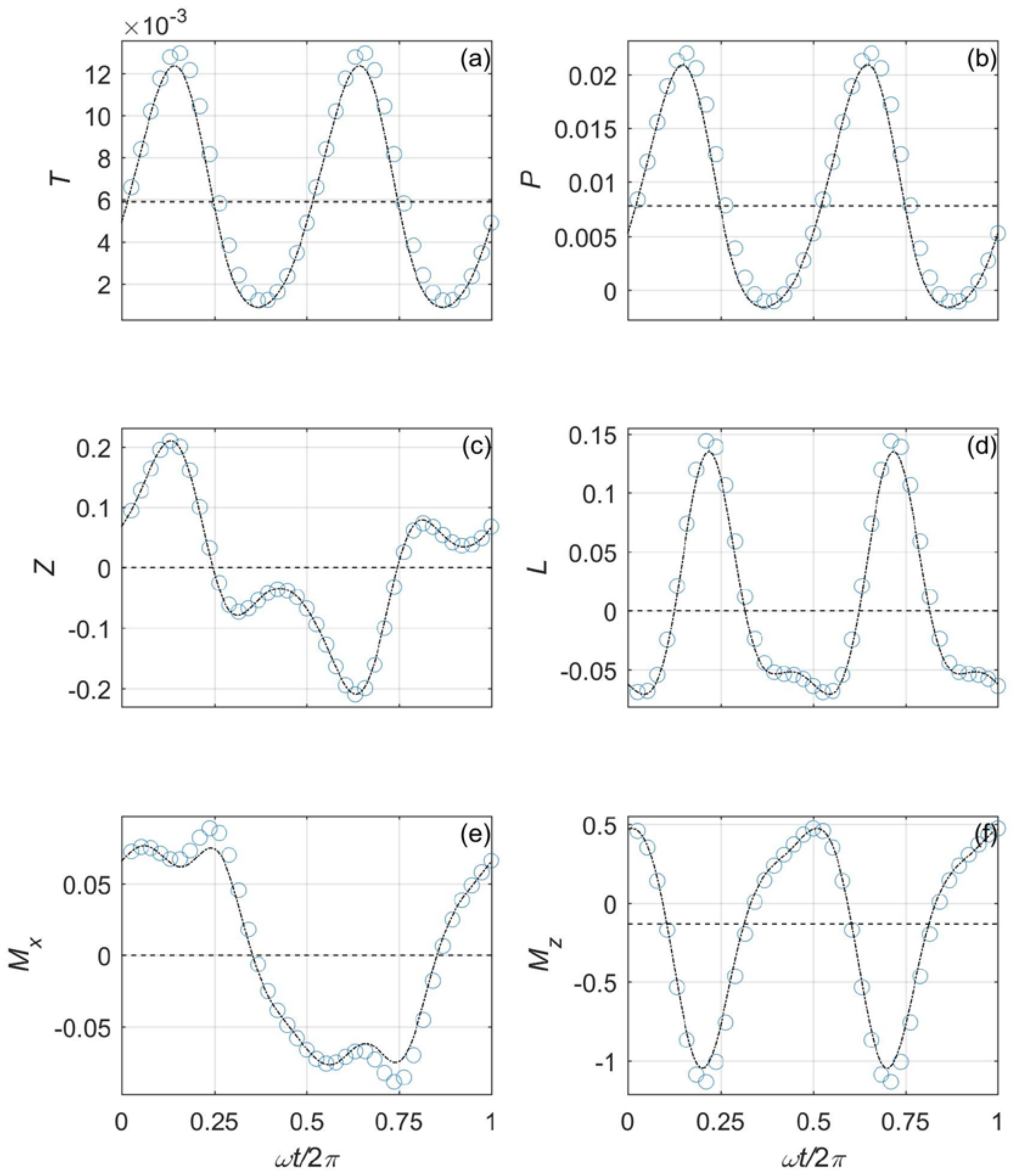

Figure S5: T, P, Z, L, $M_{x'}$ and $M_{z',t}$ over a single period. Circles mark the respective numerical simulation; dash-dot lines show equations (S8), (S11), (S10), (S9), (S12) and (S7), respectively; horizontal dashed lines mark the time-averaged values from (3.26), (3.29), (3.28), (3.27), (3.30) and (3.34) from the companion paper. Case 5 from Table S1.

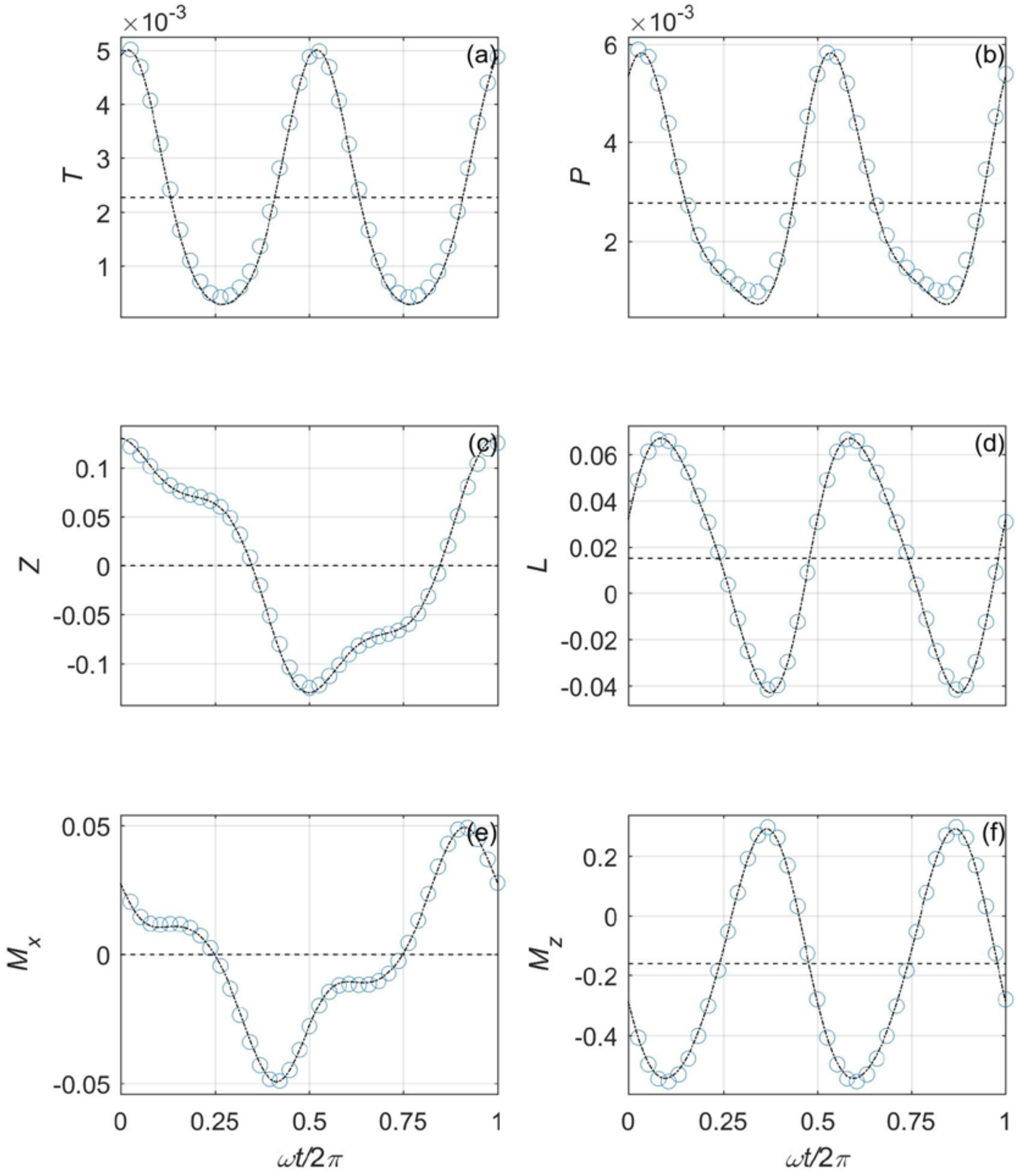

Figure S6: T, P, Z, L, $M_{x'}$ and $M_{z',t}$ over a single period. Circles mark the respective numerical simulation; dash-dot lines show equations (S8), (S11), (S10), (S9), (S12) and (S7), respectively; horizontal dashed lines mark the time-averaged values from (3.26), (3.29), (3.28), (3.27), (3.30) and (3.34) from the companion paper. Case 6 from Table S1.

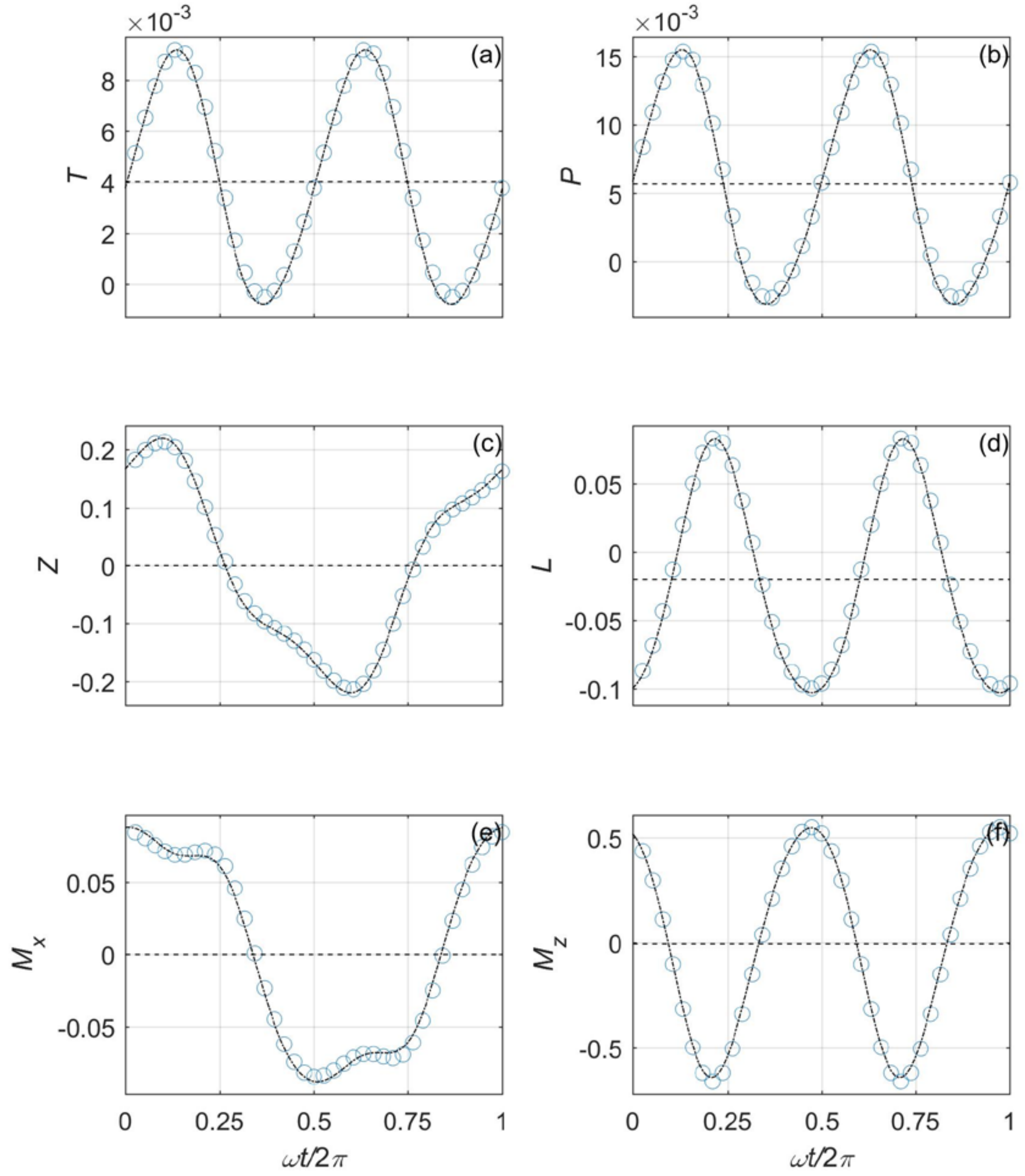

Figure S7: T, P, Z, L, $M_{x'}$ and $M_{z',t}$ over a single period. Circles mark the respective numerical simulation; dash-dot lines show equations (S8), (S11), (S10), (S9), (S12) and (S7), respectively; horizontal dashed lines mark the time-averaged values from (3.26), (3.29), (3.28), (3.27), (3.30) and (3.34) from the companion paper. Case 7 from Table S1.

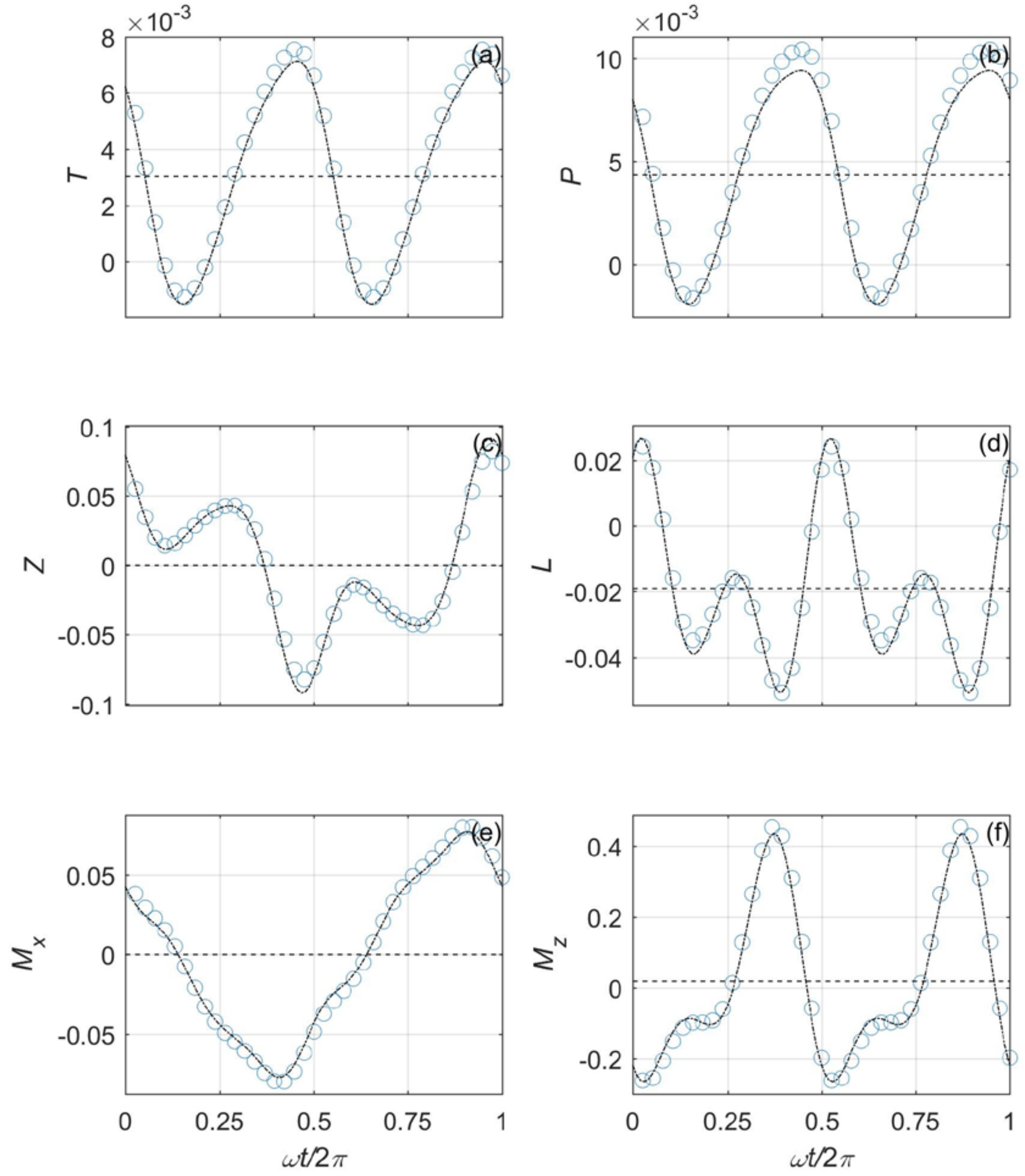

Figure S8: T, P, Z, L, $M_{x'}$ and $M_{z',t}$ over a single period. Circles mark the respective numerical simulation; dash-dot lines show equations (S8), (S11), (S10), (S9), (S12) and (S7), respectively; horizontal dashed lines mark the time-averaged values from (3.26), (3.29), (3.28), (3.27), (3.30) and (3.34) from the companion paper. Case 8 from Table S1.

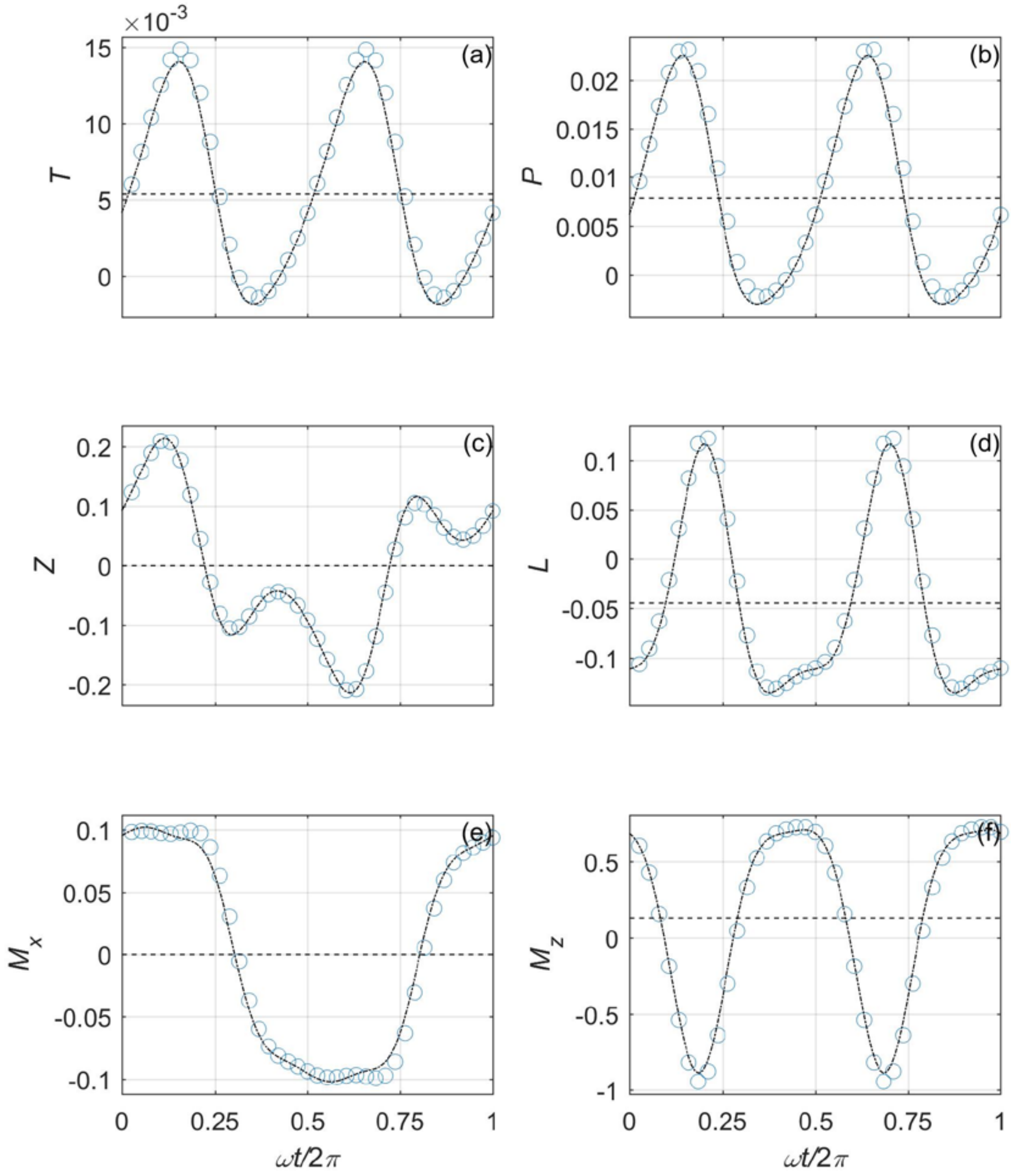

Figure S9: T, P, Z, L, $M_{x'}$ and $M_{z',t}$ over a single period. Circles mark the respective numerical simulation; dash-dot lines show equations (S8), (S11), (S10), (S9), (S12) and (S7), respectively; horizontal dashed lines mark the time-averaged values from (3.26), (3.29), (3.28), (3.27), (3.30) and (3.34) from the companion paper. Case 9 from Table S1.

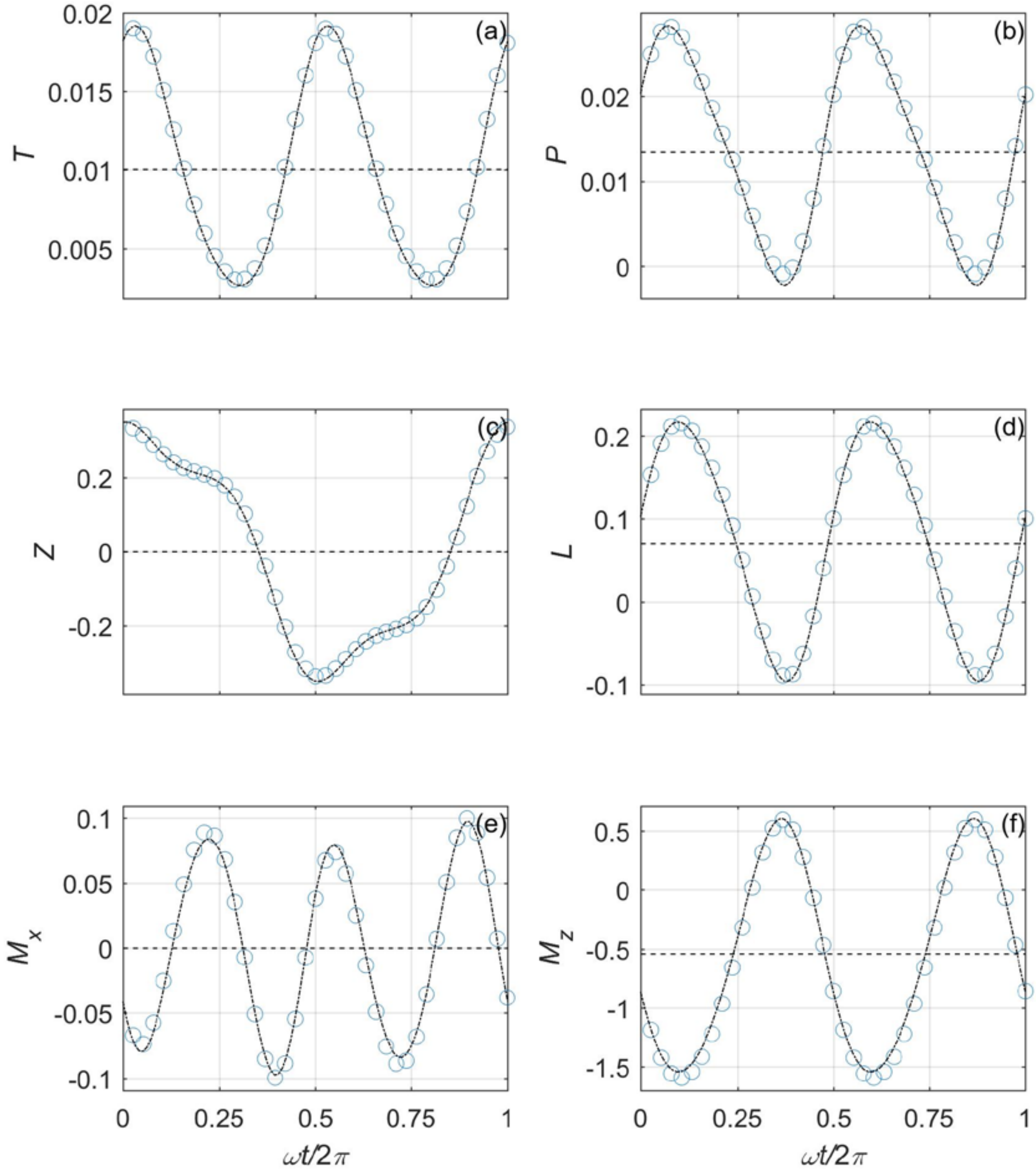

Figure S10: T, P, Z, L, $M_{x'}$ and $M_{z',t}$ over a single period. Circles mark the respective numerical simulation; dash-dot lines show equations (S8), (S11), (S10), (S9), (S12) and (S7), respectively; horizontal dashed lines mark the time-averaged values from (3.26), (3.29), (3.28), (3.27), (3.30) and (3.34) from the companion paper. Case 10 from Table S1.

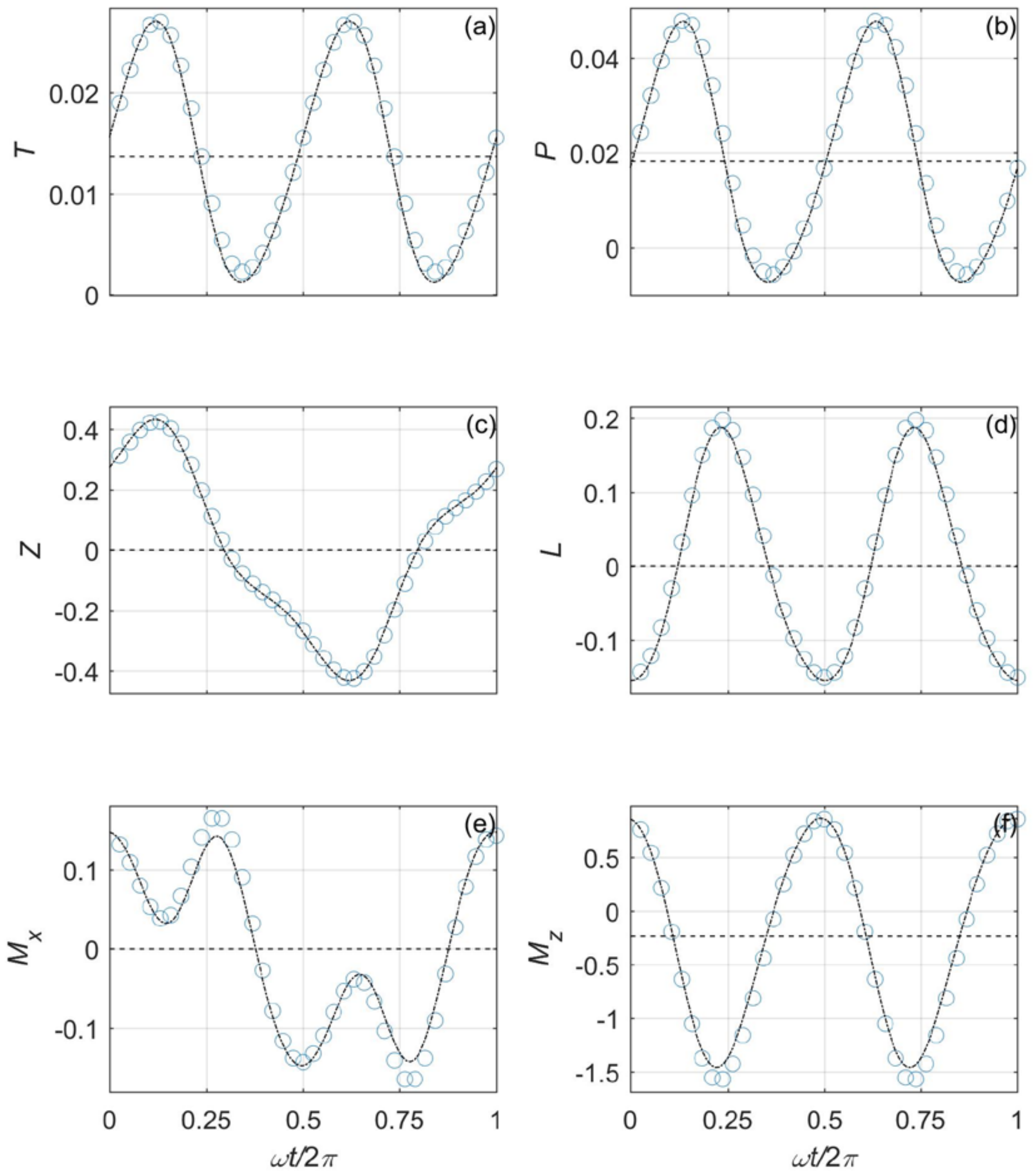

Figure S11: T, P, Z, L, $M_{x'}$ and $M_{z',t}$ over a single period. Circles mark the respective numerical simulation; dash-dot lines show equations (S8), (S11), (S10), (S9), (S12) and (S7), respectively; horizontal dashed lines mark the time-averaged values from (3.26), (3.29), (3.28), (3.27), (3.30) and (3.34) from the companion paper. Case 11 from Table S1.

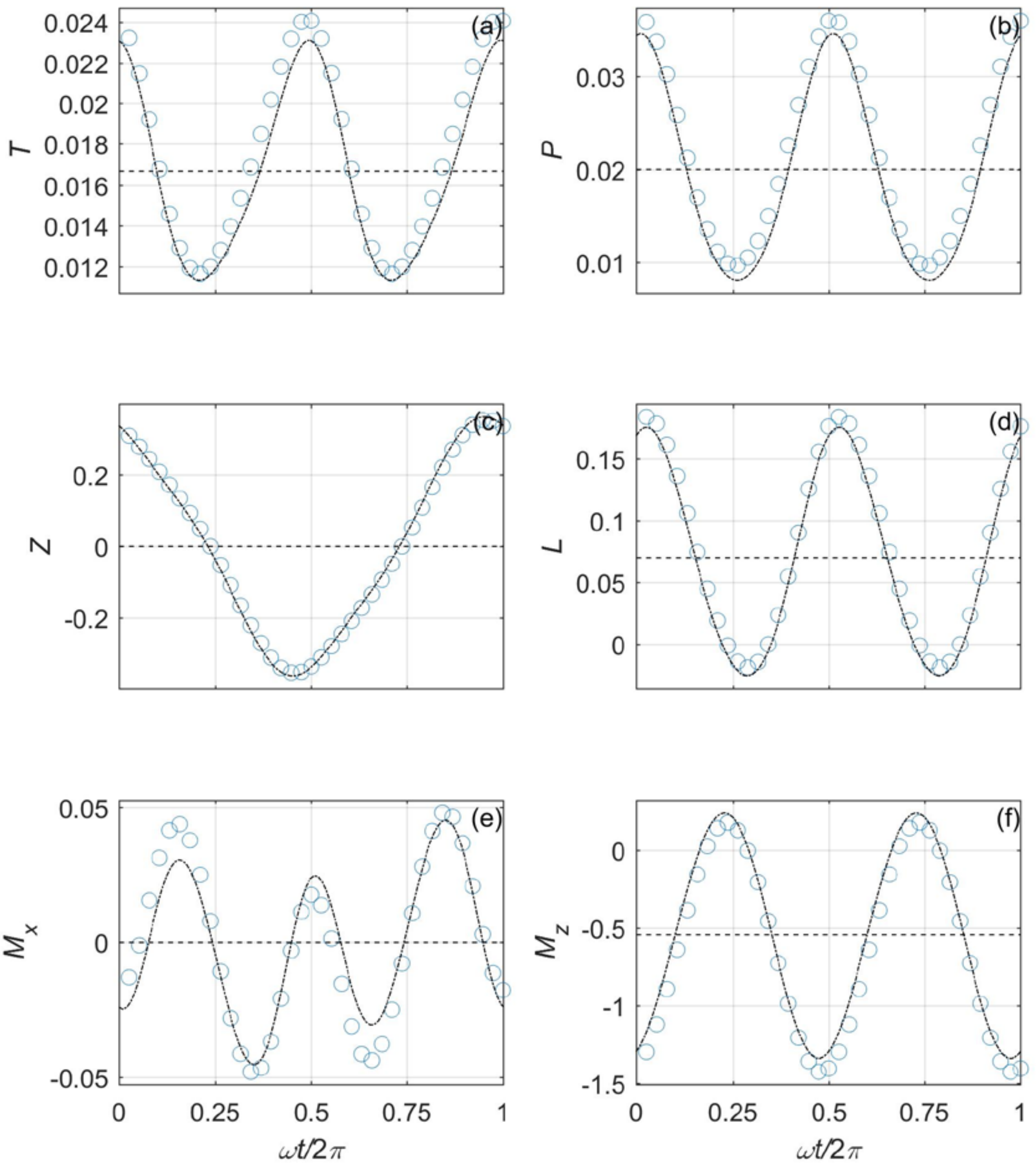

Figure S12: T, P, Z, L, $M_{x'}$ and $M_{z',t}$ over a single period. Circles mark the respective numerical simulation; dash-dot lines show equations (S8), (S11), (S10), (S9), (S12) and (S7), respectively; horizontal dashed lines mark the time-averaged values from (3.26), (3.29), (3.28), (3.27), (3.30) and (3.34) from the companion paper. Case 12 from Table S1.

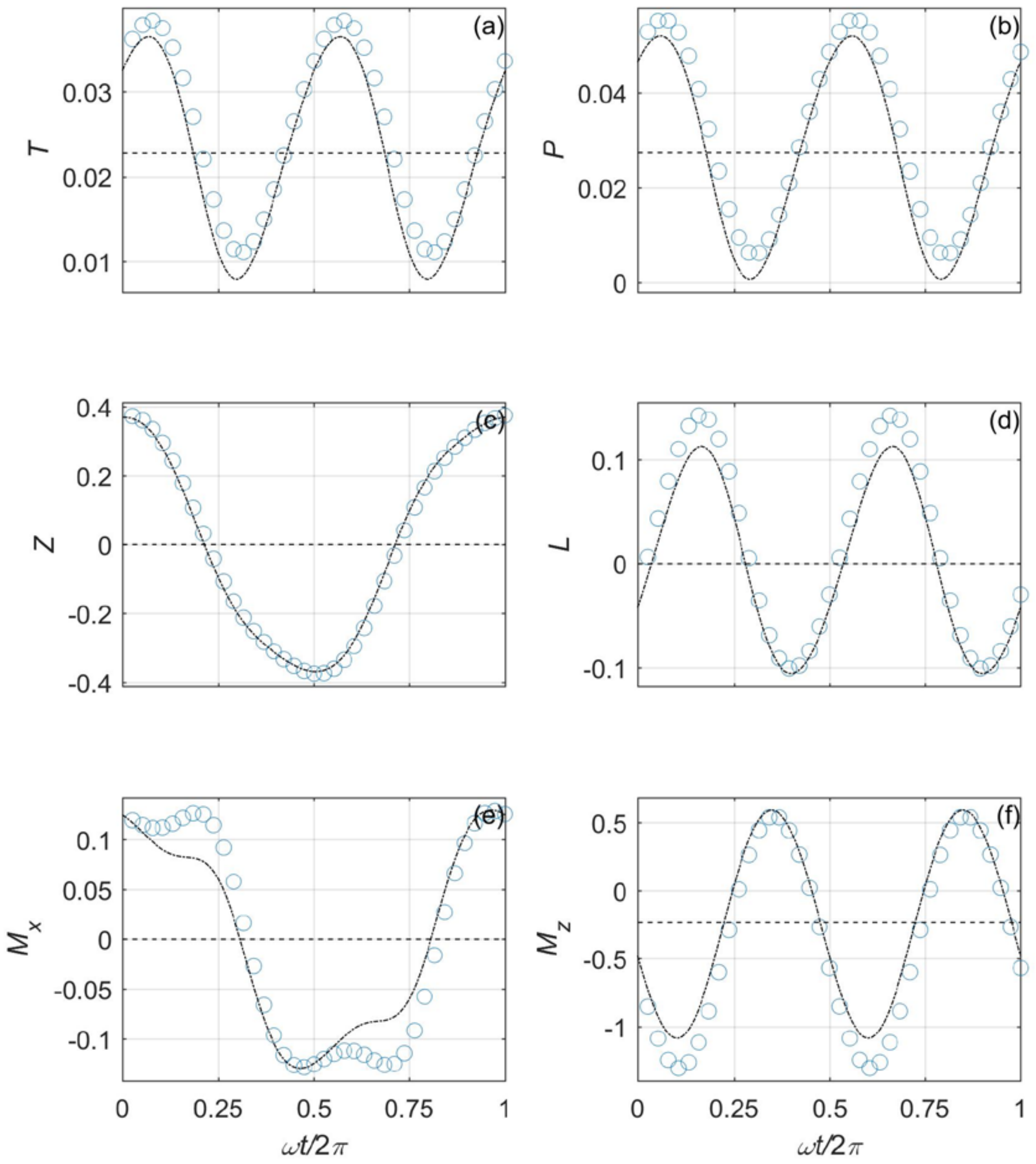

Figure S13: T, P, Z, L, $M_{x'}$ and $M_{z',t}$ over a single period. Circles mark the respective numerical simulation; dash-dot lines show equations (S8), (S11), (S10), (S9), (S12) and (S7), respectively; horizontal dashed lines mark the time-averaged values from (3.26), (3.29), (3.28), (3.27), (3.30) and (3.34) from the companion paper. Case 13 from Table S1.

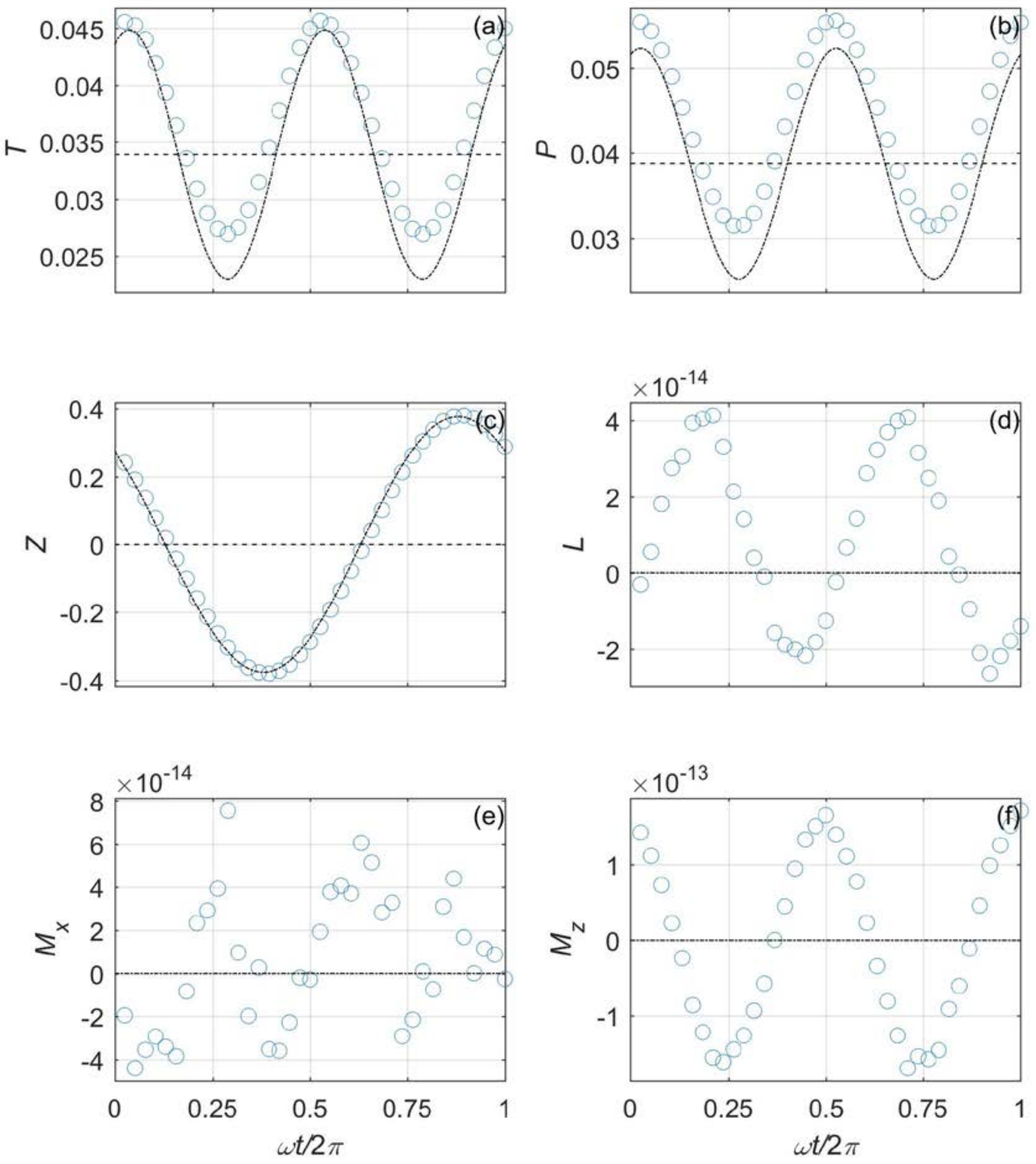

Figure S14: T, P, Z, L, $M_{x'}$ and $M_{z',t}$ over a single period. Circles mark the respective numerical simulation; dash-dot lines show equations (S8), (S11), (S10), (S9), (S12) and (S7), respectively; horizontal dashed lines mark the time-averaged values from (3.26), (3.29), (3.28), (3.27), (3.30) and (3.34) from the companion paper. Case 14 from Table S1. Note the diminutive values of L, $M_{x'}$ and $M_{z',t}$.

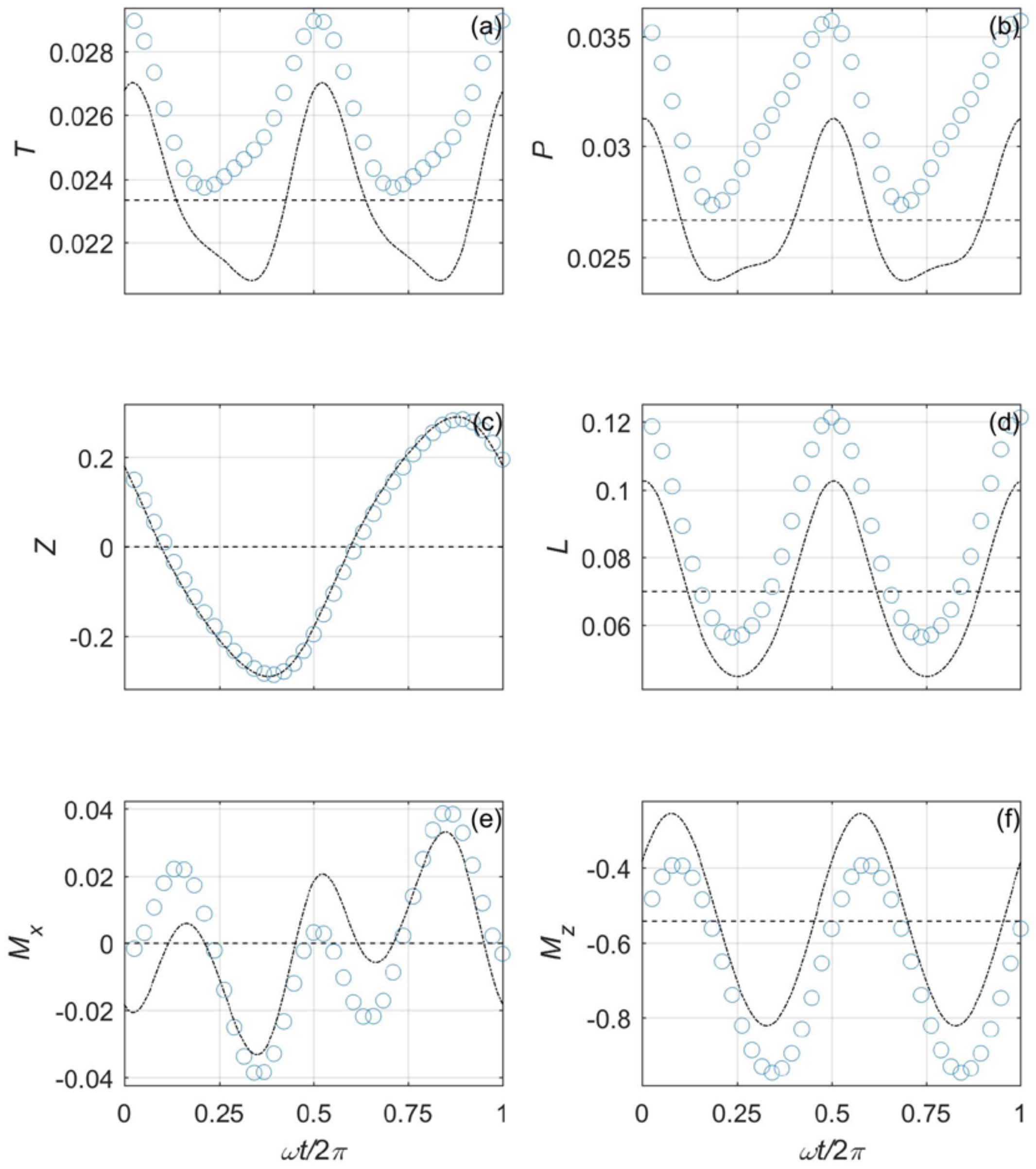

Figure S15: T, P, Z, L, $M_{x'}$ and $M_{z',t}$ over a single period. Circles mark the respective numerical simulation; dash-dot lines show equations (S8), (S11), (S10), (S9), (S12) and (S7), respectively; horizontal dashed lines mark the time-averaged values from (3.26), (3.29), (3.28), (3.27), (3.30) and (3.34) from the companion paper. Case 15 from Table S1.

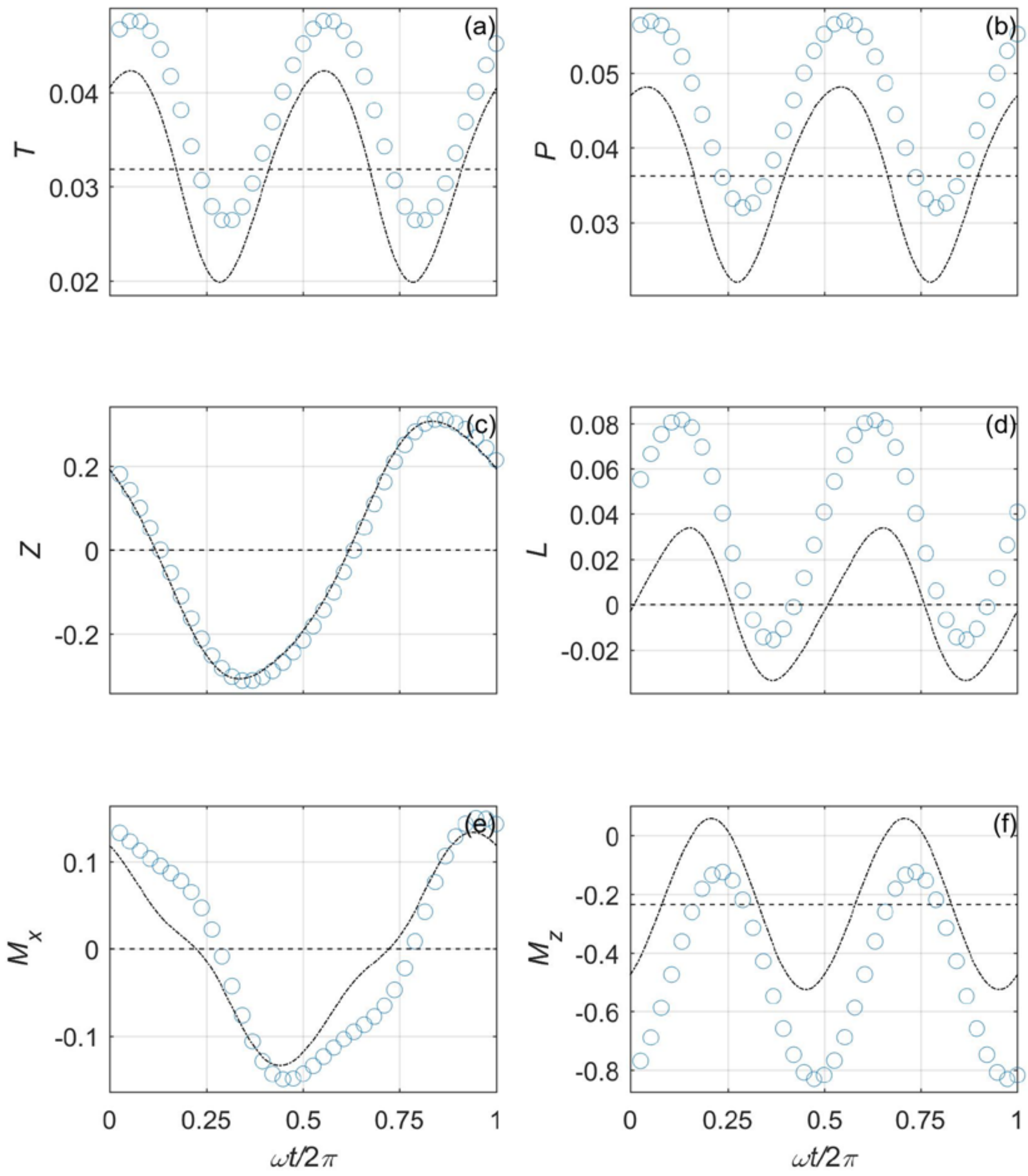

Figure S16: T, P, Z, L, $M_{x'}$ and $M_{z',t}$ over a single period. Circles mark the respective numerical simulation; dash-dot lines show equations (S8), (S11), (S10), (S9), (S12) and (S7), respectively; horizontal dashed lines mark the time-averaged values from (3.26), (3.29), (3.28), (3.27), (3.30) and (3.34) from the companion paper. Case 16 from Table S1.

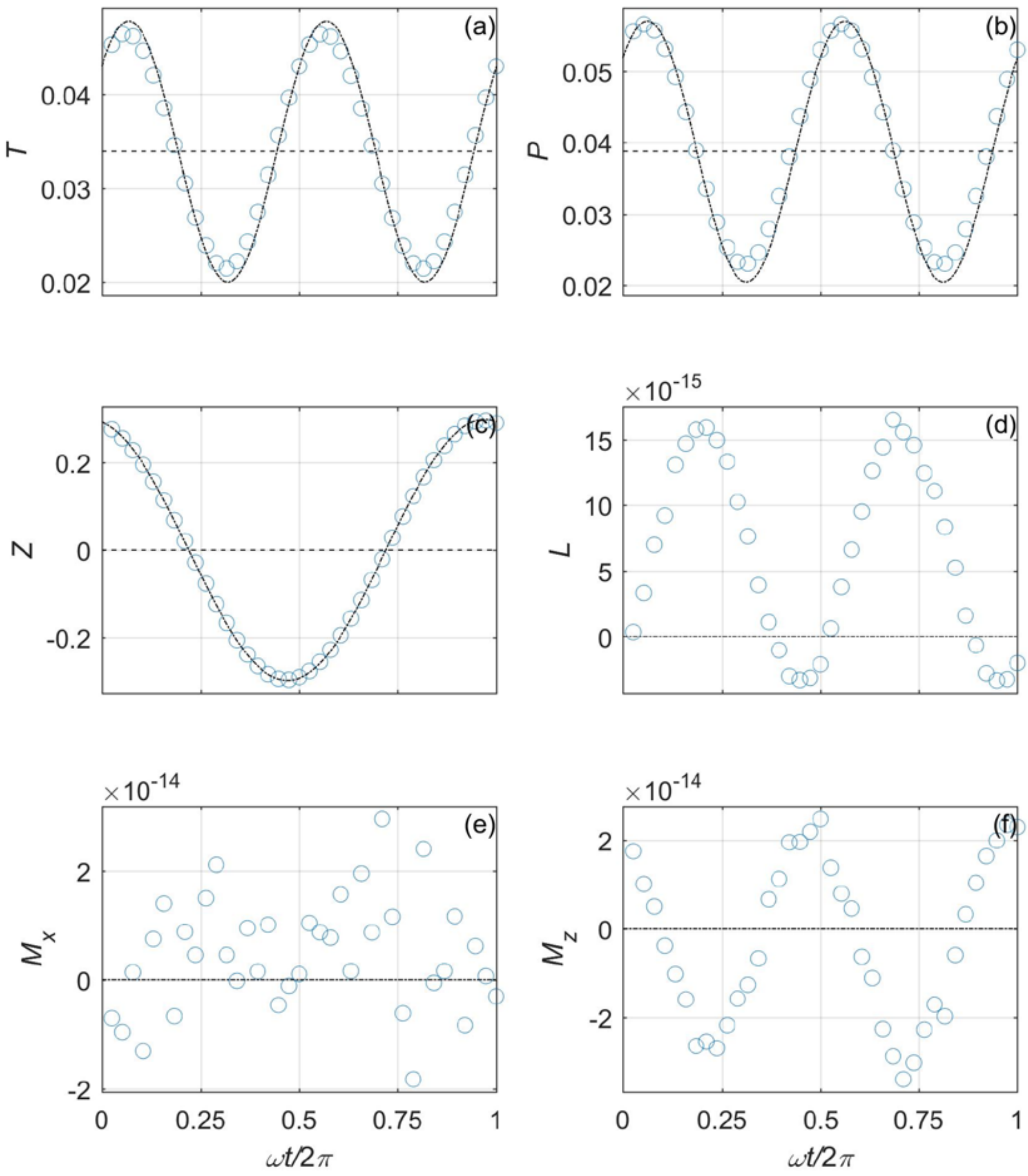

Figure S17: T, P, Z, L, $M_{x'}$ and $M_{z',t}$ over a single period. Circles mark the respective numerical simulation; dash-dot lines show equations (S8), (S11), (S10), (S9), (S12) and (S7), respectively; horizontal dashed lines mark the time-averaged values from (3.26), (3.29), (3.28), (3.27), (3.30) and (3.34) from the companion paper. Case 17 from Table S1. Note the diminutive values of L, $M_{x'}$ and $M_{z',t}$.

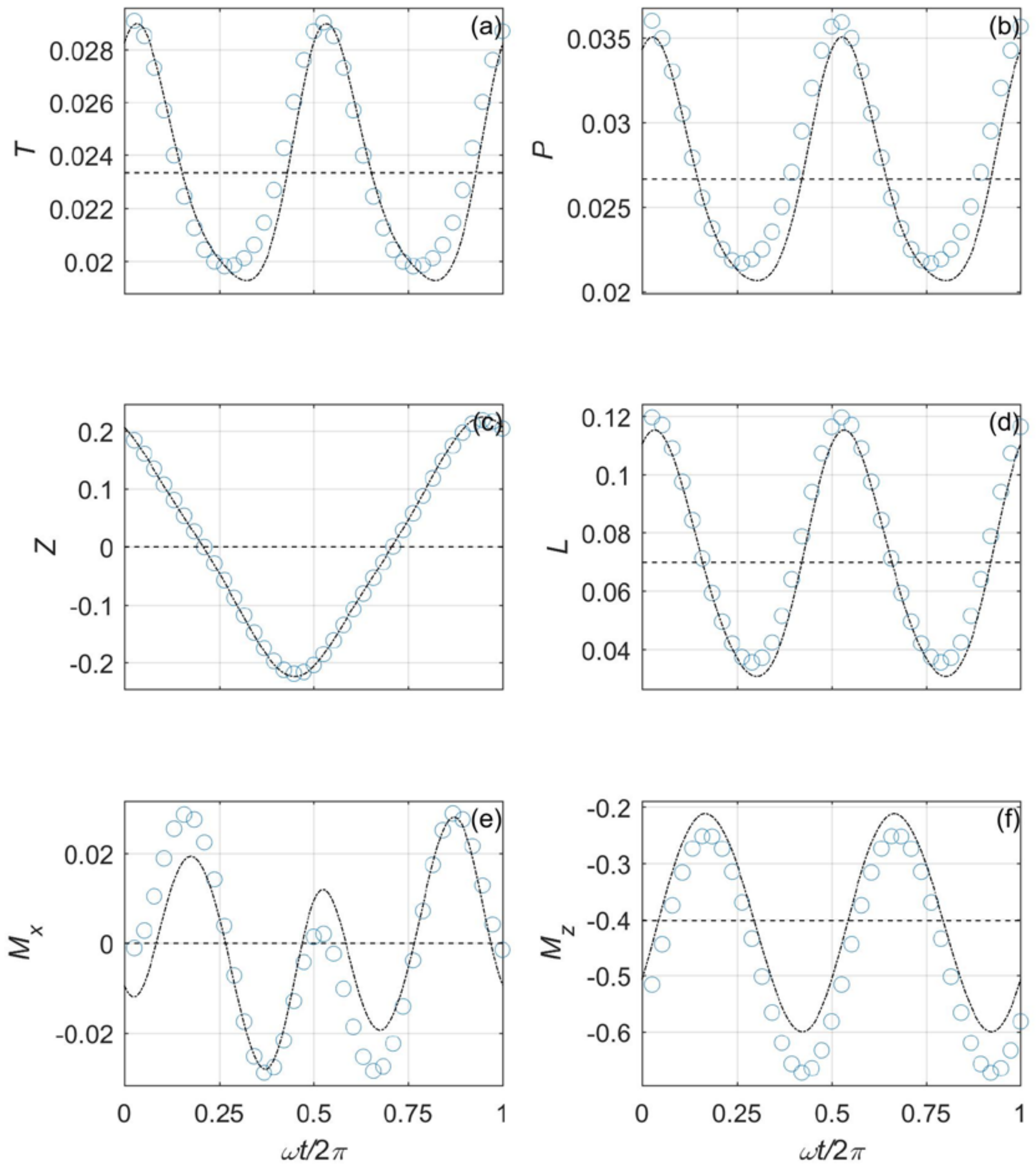

Figure S18: T, P, Z, L, M_x and M_z over a single period. Circles mark the respective numerical simulation; dash-dot lines show equations (S8), (S11), (S10), (S9), (S12) and (S7), respectively; horizontal dashed lines mark the time-averaged values from (3.26), (3.29), (3.28), (3.27), (3.30) and (3.34) from the companion paper. Case 18 from Table S1.

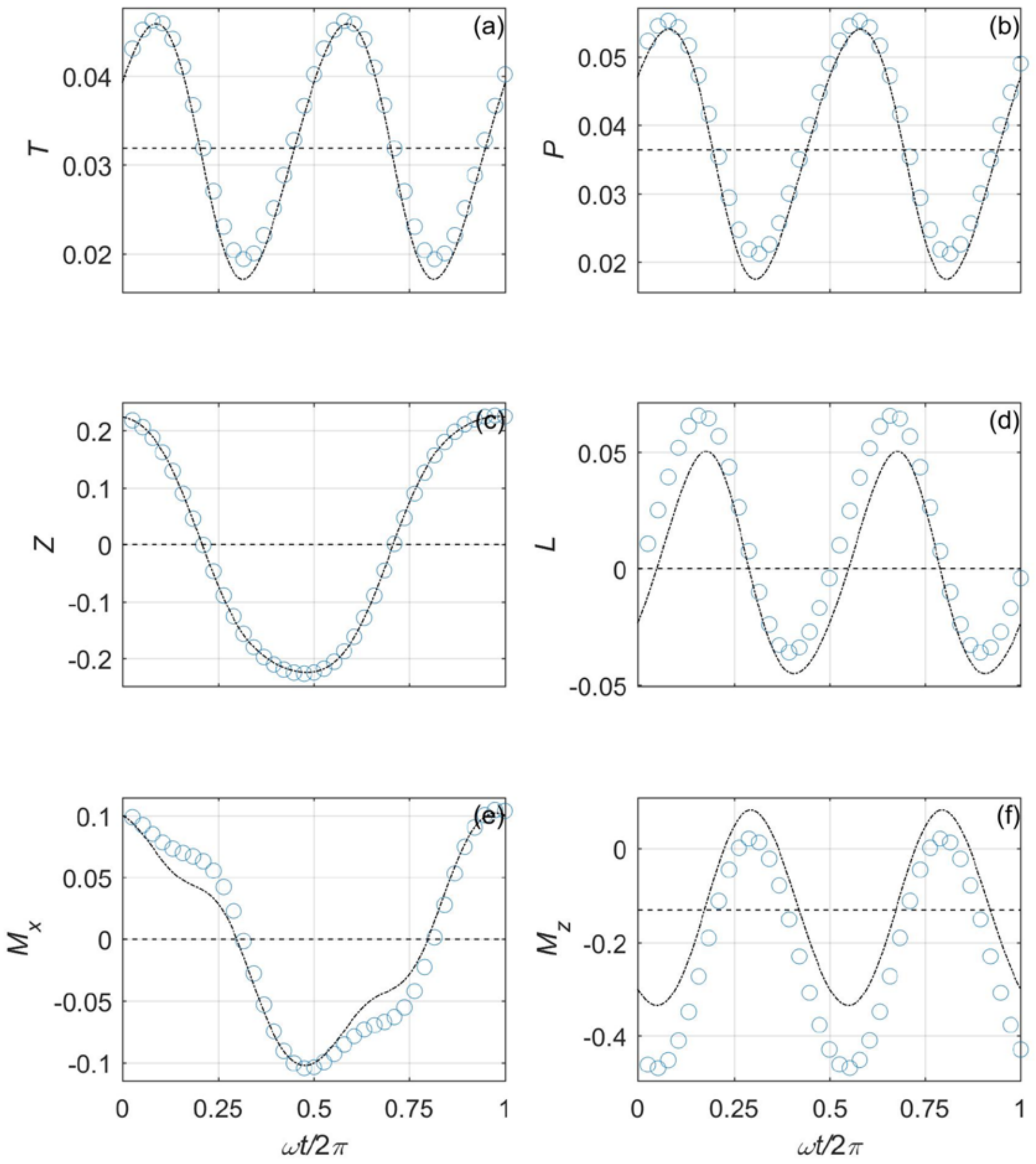

Figure S19: T, P, Z, L, M_x and M_z over a single period. Circles mark the respective numerical simulation; dash-dot lines show equations (S8), (S11), (S10), (S9), (S12) and (S7), respectively; horizontal dashed lines mark the time-averaged values from (3.26), (3.29), (3.28), (3.27), (3.30) and (3.34) from the companion paper. Case 19 from Table S1.
